# A Decline in New HIV Infections in South Africa: Estimating HIV Incidence from Three National HIV Surveys in 2002, 2005 and 2008

**DOI:** 10.1371/journal.pone.0011094

**Published:** 2010-06-14

**Authors:** Thomas M. Rehle, Timothy B. Hallett, Olive Shisana, Victoria Pillay-van Wyk, Khangelani Zuma, Henri Carrara, Sean Jooste

**Affiliations:** 1 Human Sciences Research Council, Cape Town, South Africa; 2 Imperial College London, London, United Kingdom; Erasmus University Rotterdam, Netherlands

## Abstract

**Background:**

Three national HIV household surveys were conducted in South Africa, in 2002, 2005 and 2008. A novelty of the 2008 survey was the addition of serological testing to ascertain antiretroviral treatment (ART) use.

**Methods and Principal Findings:**

We used a validated mathematical method to estimate the rate of new HIV infections (HIV incidence) in South Africa using nationally representative HIV prevalence data collected in 2002, 2005 and 2008. The observed HIV prevalence levels in 2008 were adjusted for the effect of antiretroviral treatment on survival. The estimated “excess” HIV prevalence due to ART in 2008 was highest among women 25 years and older and among men 30 years and older. In the period 2002–2005, the HIV incidence rate among men and women aged 15–49 years was estimated to be 2.0 new infections each year per 100 susceptible individuals (/100pyar) (uncertainty range: 1.2–3.0/100pyar). The highest incidence rate was among 15–24 year-old women, at 5.5/100pyar (4.5–6.5). In the period 2005–2008, incidence among men and women aged 15–49 was estimated to be 1.3/100 (0.6–2.5/100pyar), although the change from 2002–2005 was not statistically significant. However, the incidence rate among young women aged 15–24 declined by 60% in the same period, to 2.2/100pyar, and this change was statistically significant. There is evidence from the surveys of significant increases in condom use and awareness of HIV status, especially among youth.

**Conclusions:**

Our analysis demonstrates how serial measures of HIV prevalence obtained in population-based surveys can be used to estimate national HIV incidence rates. We also show the need to determine the impact of ART on observed HIV prevalence levels. The estimation of HIV incidence and ART exposure is crucial to disentangle the concurrent impact of prevention and treatment programs on HIV prevalence.

## Introduction

Worldwide, South Africa has the highest number of HIV infected individuals, with about 5.3 million people living with HIV/AIDS, representing a quarter of the burden of HIV infection in sub-Saharan Africa [1], [2]. The National HIV&AIDS and STI Strategic Plan 2007–2011 has begun to address this enormous challenge for South Africa [3]. The two primary goals of the plan are to reduce the HIV incidence rate by 50% and to expand the access to antiretroviral treatment (ART) to 80% of people who need it. South Africa's scale-up of ART is unmatched in the world, increasing from only 32,895 people on ART by January 2005 to 871,914 people enrolled in the public sector program by July 2009 (official statistics of the Department of Health, South Africa, July 2009).

The interpretation of HIV prevalence trends in South Africa is increasingly complex as the epidemic matured and prevention and treatment programmes are implemented at the same time. Increased access to ART has increased the survival time of people living with HIV, with the effect that HIV prevalence is expected to increase in the age groups who are predominantly receiving ART. The estimation of HIV incidence is crucial in this evolving scenario in order to disentangle the impact of prevention and treatment programs on HIV prevalence.

Recently, a new method has been described that can estimate HIV incidence by comparing prevalence data in two cross-sectional prevalence surveys [4]. In this method, the difference in prevalence between the two surveys is equal to effect of HIV/AIDS-related deaths and new infections occurring during the inter-survey interval; and with an estimate of AIDS deaths, incidence can be deduced. This method has been thoroughly validated using simulated data and through comparison of its estimates with real measurements of incidence in several community-based cohort studies in Africa [4]. The method has since been used to estimate incidence in Tanzania and Zambia where two cross-sectional prevalence surveys have been conducted [5]. The validity of the estimate hinges on there being several successive comparable surveys measuring national HIV prevalence in the general population.

Estimating incidence in this way is ideally suited to South Africa, since there have been three large nationally representative household-based surveys, in 2002, 2005 and 2008 [6]. Furthermore, the survey in 2008 identified individuals on ART by means of testing HIV positive samples for the presence of antiretroviral drugs, so that the effect of treatment on prevalence could be accounted for adequately. These data can thus be used to derive two estimates for the 2002–2005 and 2005–2008 inter-survey intervals and identifying changes in incidence over time in recent years.

Our objectives are (i) to attempt to quantify the impact of ART on HIV prevalence following the rapid scale-up of access to treatment, (ii) to calculate HIV incidence for two inter-survey intervals and determine changes in incidence in recent years in South Africa, and (iii) to examine changes in key behavioral indicators collected in the three surveys among female youth aged 15–24 years.

## Methods

### Survey data

**Figure 1:**
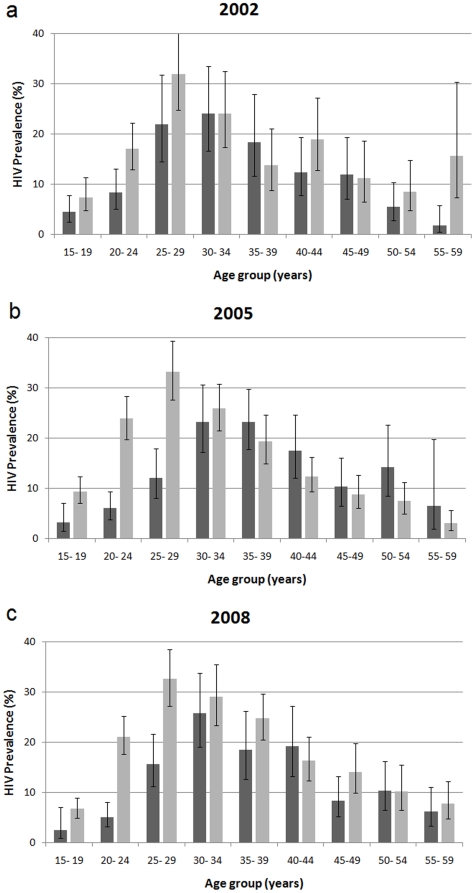
HIV prevalence in South Africa in (a) 2002, (b) 2005 and (c) 2008. The dark bars show the prevalence among men and the lighter bars show the prevalence among women. Source: Human Science Research Council Surveys [6].

Three national HIV household surveys have been conducted in South Africa, the first in 2002 followed by surveys in 2005 and 2008. Figure 1 shows the HIV prevalence profiles in the three surveys by age and sex. All three surveys applied a multi-stage, stratified sampling approach. The sampling frames for the surveys were based on a master sample consisting of 1 000 enumerator areas (EA) used by Statistics South Africa for the 2001 census. An updated master sample had to be developed for the 2008 survey in order to reflect the changing socio-demographic profile of the country. The selection of EAs was stratified by province and locality type. Locality types were identified as urban formal, urban informal, rural formal (including commercial farms), and rural informal. In the formal urban areas, race was also used as a third stratification variable (based on the predominant race group in the selected EA at the time of the 2001 census). The allocation of EAs to different stratification categories was disproportionate with over-sampling of EAs in areas that were dominated by Indian, Coloured or White race groups to ensure that the minimum required sample size in those smaller race groups was obtained [6].

The selected 1000 EAs formed the primary sampling units. Visiting points, or households were used as secondary sampling units. Within each household eligible individuals selected for the survey represented the ultimate sampling unit. Weighting procedures taking into account the complex sampling design and HIV testing non-response produced a final sample representative of the population in South Africa for the main reporting domains sex, age, race, locality type and province. Individual sample weights were benchmarked using the mid-year population estimates provided by Statistics South Africa for the respective survey years 2002, 2005 and 2008.

Structured questionnaires were used to collect demographic, social and behavioural data. Oral mucosal transudate specimens (n = 8 428) were used in 2002, while in 2005 (n = 15 851) and 2008 (n = 15 031) dried blood spot (DBS) specimens were used for HIV testing. The mid-points of data collection in the surveys were June 2002, February 2005 and September 2008. HIV testing coverage and non-response were analyzed for the main reporting domains. Our detailed analysis comparing HIV risk-associated characteristics in survey participants who were interviewed and tested with those who were interviewed but refused HIV testing suggests that the HIV survey results were not biased due to HIV testing refusal [6].

### Detection of antiretroviral drugs

In the 2008 survey, the presence of antiretroviral drugs (ARVs) in HIV positive DBS samples was confirmed by means of High Performance Liquid Chromatography (HPLC) coupled to Tandem Mass Spectrometry. Qualitative detection of Lopinavir, Ritonavir, Nevirapine, Efavirenz, Indinavir, Saquinavir, Zidovudine, Lamivudine and Stavudine in DBS samples was carried out by a validated method using minor modifications of the method used by Koal et al. [7]. Antiretroviral drugs were extracted from the DBS samples with 80% methanol and 20% 0.2M Zinc Sulphate containing an internal standard. HPLC was carried out on a Phenomonex Fusion RP column (5×2×4µm) using a methanol/10 mM ammonium acetate gradient to effect elution. Detection of antiretroviral drugs was carried out using an Applied Biosystems API 4000 tandem mass spectrometer in the multiple reaction monitoring (MRM) detection mode for each drug using appropriate MRM transitions. Blank and quality control cutoff samples were included with each run. The limit of detection for each drug was set at 50 ng/ml, a sensitivity set point which is normally applied for quantitative monitoring of drug levels in the blood. Values detected above this limit were considered as positive and those below as negative.

### Analytic Method

Our method for estimating incidence from two cross-sectional prevalence measurements [4] is based on the synthetic cohort principle, whereby we assume that individuals of age 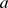 in the first survey will be represented by individuals aged 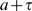 in the second survey, where 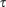 is the interval between surveys, even though the surveys do not include the same individuals. The change in HIV prevalence among individuals aged 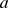 in the first survey and 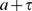 in the second survey can be attributed to incident infections and AIDS deaths. By finding an approximate value for the rate of AIDS deaths, based on the observed distribution of survival after infection [8], HIV incidence for that age-group can be estimated. Thus, using prevalence data collected in 2002 and 2005 an estimate for incidence in the period 2002–2005 is found; and by using prevalence data collected in 2005 and 2008, an estimate for incidence in the period 2005–2008 is found. The interval between the surveys (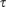) was 2 years 8 months for 2002–2005 and 3 years 7 months for 2005–2008, but since respondents reported only their age in years at last birthday (rather than date of birth), it was necessary to interpolate prevalence in the exact synthetic cohorts.

To allow for the effect of antiretroviral treatment in 2008, which can prolong survival with HIV, the observed prevalence values were changed to reflect HIV prevalence if no treatment were available. This involves subtracting the proportion of HIV-infected people who were alive in the survey that would have died without treatment. This is closely related to the number of people receiving treatment, but is actually lower because some of those on treatment would have survived for some time even without treatment. Thus, data on the scale-up of antiretroviral treatment are used (Figure S1 in the Technical Appendix S1), and it is assumed that treatment is typically initiated one year before expected AIDS death [9]. This simple approach allows the previously validated methods for approximation HIV-related mortality (for those not on ART) to be used correctly [5]. For the point estimates, we assume that the mortality rate in the first years on treatment is 10% per year. Further information on the method is available in the technical appendix. This part of the method had been validated with simulated data but not real cohort data.

We use bootstrapping (repeating the incidence estimation with prevalence values from simulated draws of a binomial model representing the survey data) to determine how random errors in the estimates of HIV prevalence in the survey propagate to errors in the estimates of HIV incidence [10]. The uncertainty in the estimates of incidence in 2005–2008 also includes the effect of mortality during the first years on ART being lower (5% per year) or higher (15% per year) than the rate assumed for the point estimate. The results do not explicitly account for uncertainty in the survival distribution following infection.

## Results

In order to draw comparison between the prevalence estimates obtained in 2002 and 2005, when a negligible number of individuals were on treatment, with prevalence in 2008, when 17.5% of HIV-infected survey participants aged 15–49 years tested positive for antiretroviral treatment, we make an adjustment to the data in 2008. This adjustment removes those individuals that are HIV-infected and currently receiving treatment and are alive due to the effects of treatment. Assuming that the trend in the numbers on treatment in the public sector reflects the overall increase in treatment, and that treatment is typically initiated approximately one year before individuals would otherwise die [9], [11], [12] and that individuals starting treatment suffer a 10% annual mortality rate in the first years of treatment [12], [13], we estimate that 58% of those receiving treatment in 2008 are alive due to ART. The remaining 42% would therefore be expected to be alive and present in the survey, even if they had not been started on treatment.

**Figure 2:**
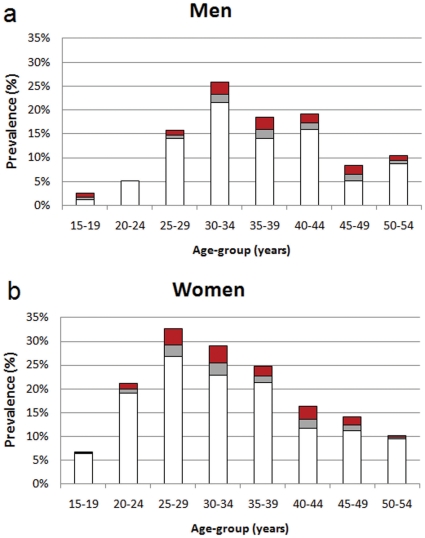
The impact of antiretroviral therapy on HIV prevalence in 2008 for (a) men and (b) women. In each panel, the overall height of the bars shows HIV prevalence; the white part shows those HIV-infected but not on treatment; the grey part shows those on treatment but who would be still alive without treatment; and, the red part shows those on treatment who are alive due to treatment. The estimate of the proportion alive due to treatment is based on assumptions that treatment is initiated one year before when individuals would otherwise die, and that individuals on treatment suffer a 10% annual mortality rate in first years of treatment.

Figure 2 shows the measured HIV prevalence level among men and women in 2008, decomposed into those that are infected but not on treatment (white areas), those on treatment that would otherwise be alive (grey areas), and those alive due to treatment (red areas). We thus calculate the increase (‘excess’) HIV prevalence that is caused by antiretroviral treatment. Overall, prevalence in 2008 among men and women aged 15–49 years was 16.9%. We estimate that the excess prevalence due to antiretroviral treatment in this age group is 1.7%, and that prevalence in 2008 in the absence of treatment would be 15.2%. Compared to the estimate of prevalence in the same group in 2005 (16.2%), the unadjusted value of prevalence in 2008 indicates a small increase, whereas the adjusted value suggests a noteworthy decline.

Table 1 shows the excess prevalence by age and sex. The excess HIV prevalence is highest among women 25 years and older and among men 30 years and older. For example, among women aged 30–34 years, the observed HIV prevalence in 2008 is 29.1%, but ART accounts for an excess prevalence of 3.6%, meaning that the adjusted value of HIV prevalence without ART is 25.5%.

**Table 1:** Impact of antiretroviral therapy on HIV prevalence in South Africa 2008.

Age group (years)	HIV Prevalence in 2005 (%)	HIV Prevalence in 2008 (%)	Excess HIV Prevalence due to ART in 2008 (%)^1^	Adjusted HIV Prevalence without ART in 2008 (%)^2^
**Men and Women**				
**15–49**	16.2	16.9	1.7	15.2
**Men**				
**15–19**	3.2	2.5	0.8	1.7
**20–24**	6.0	5.1	0.0	5.1
**25–29**	12.1	15.7	0.9	14.8
**30–34**	23.3	25.8	2.5	23.3
**35–39**	23.3	18.5	2.6	15.9
**40–44**	17.5	19.2	2.0	17.2
**45–49**	10.3	8.4	1.9	6.5
**50–54**	14.2	10.4	1.0	9.4
**15–49**	11.7	11.6	1.3	10.3
**Women**				
**15–19**	9.4	6.7	0.2	6.5
**20–24**	23.9	21.1	1.2	19.9
**25–29**	33.3	32.7	3.4	29.3
**30–34**	26.0	29.1	3.6	25.5
**35–39**	19.3	24.8	2.1	22.7
**40–44**	12.4	16.3	2.7	13.6
**45–49**	8.7	14.1	1.7	12.4
**50–54**	7.4	10.2	0.3	9.9
**15–49**	20.2	21.3	2.1	19.2

1 The proportion of individuals who are alive because they are currently on ART and who would be dead otherwise.

2 Estimated HIV prevalence if there was no ART in 2008.

**Figure 3:**
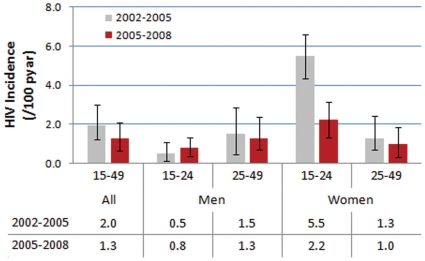
Estimates of HIV incidence rate in South Africa, 2002–2005 (light grey bars) and 2005–2008 (dark red bars), using cross-sectional HIV prevalence data collected in 2002, 2005 and 2008. The error bars for the 2002–2005 estimates show the 95% uncertainty interval due to measurement errors in the prevalence data; the error bars for the 2005–2008 estimates show the uncertainty due to measurement error and the use of alternate assumptions for the ART adjustment (described in the text).

The overall estimates of incidence in the periods 2002–2005 and 2005–2008 are shown in Figure 3. The overall estimate of HIV incidence in the period 2002–2005 for men and women aged 15–49 years is 2.0 per 100 person-years at risk (pyar) (95% uncertainty range: 1.2–3.0). In 2002–2005, incidence was much higher among women (2.8/100 pyar (2.1–3.9)) than men (1.0/100 pyar (0.3–2.0)). Incidence was ten times higher among young women aged 15–24 years (5.5/100 (4.3–6.6)) than men the same age (0.5/100 (0.1–1.1)).

A lower HIV incidence rate of 1.3/100 pyar (0.6–2.1) was estimated for women and men aged 15–49 years in the period 2005–2008. Overall, among adults aged 15–49, incidence declined by 35% between the two inter-survey periods 2002–2005 and 2005–2008. This change did not reach statistical significance, however. The decline overall was mostly due to changes in incidence among young women aged 15–24, among whom there was a statically significant 60% reduction in incidence to 2.2/100 pyar (1.3–3.1). Incidence among men and women aged 25–49 years in 2005–2008 was similar to the estimates for the period 2002–2005.

**Figure 4:**
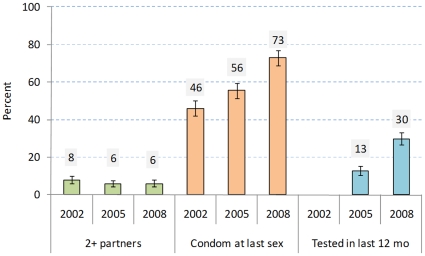
Key behavioural indicators among women aged 15–24 year in the 2002, 2005 and 2008 surveys. Indicators are: fraction reporting two or more partnerships in the last 12 months, fraction reporting condom use at last sex, and fraction that reported having an HIV test in the last 12 months. The question on testing in the last 12 months was not asked in 2002.

In Figure 4 we focus our analysis of behavioural trends on the female youth population since an incidence reduction was mainly found among young women aged 15–24. While the percentage of female youth reporting more than one sexual partner in the past 12 month did not essentially change from 2002 to 2008, very significant changes were observed in condom use at last sex and being tested for HIV. Self reported condom use increased from 46.1% in 2002 to 55.7% in 2005 and 73.1% in 2008. There was also a substantial increase in the proportion of young women who tested for HIV in the last 12 months, from 12.9% in 2005 to 29.8% in 2008. By 2008, more than half of 15–24 year-old women (52.7% (49.6–55.9%)) had ever been tested for HIV, compared with only one in eight (13.2% (10.8–16.2%)) just six years previously in 2002. We also examined other variables such as sexual debut, secondary abstinence, and intergenerational sex, and found no substantive changes over the three surveys.

## Discussion

In this analysis we have used a robust mathematical method for estimating HIV incidence in South Africa using three measures of HIV prevalence in large, nationally-representative household surveys. The mathematical method has been shown to reliably detect the level and age-pattern of HIV incidence and has previously been validated by comparing the derived estimates to ‘gold-standard’ estimates of HIV incidence in cohort studies [5]. The availability of survey data collected in 2002, 2005 and 2008 allows, for the first time, a comparison of incidence estimates for two inter-survey intervals. The data affords estimates of the average HIV incidence rate – the number of new infections occurring each year among 100 people susceptible individuals – in the periods 2002–2005 and 2005–2008 and would be comparable to the measure derived from a national HIV incidence cohort study over these intervals. Such a national incidence cohort does not exist for obvious reasons, including cost, feasibility, sampling biases and ethical considerations. However, other independent estimates of HIV incidence using a laboratory-based method for recent infection and the ASSA model suggested similar levels of incidence in 2005 [14], [15].

Our estimate of incidence highlight that young women in South Africa continue to face the highest risk of HIV infection of any demographic group. However, our results also indicate that incidence among young women has substantially reduced in recent years. There is no strong indication of a reduction in incidence among men or among older women. Nevertheless, the incidence decline among young women has resulted in a reduction in incidence among adults in South Africa overall by 35% between 2002–2005 and 2005–2008, although this was not statistically significant. Despite this encouraging development, there is no reason to become complacent. 1.3% of all uninfected South African adults, including 2.2% of uninfected young women aged 15–24 years, became newly infected in the last year. These incidence levels in the general population need to be halved in order to meet the 2011 target of the National Strategic Plan [3].

An important factor in interpreting HIV prevalence data in the era of ART is that prevalence can increase while HIV incidence is unchanged, purely as a result of ART prolonging survival of HIV-infected individuals. This means that records of prevalence remaining constant could mask real declines in incidence [16]. For this reason, we adjusted the 2008 measurement of prevalence to remove the effect of ART. This process introduces more uncertainty in the estimates of incidence since the calculation depends on the mean time of treatment initiation and rates of mortality in the first years of treatment, for which there are no direct empirical measurements in South Africa.

To address this, we made the adjustment and the estimation of incidence under a range of assumptions to quantify the uncertainty due to this approach (indicated in the error bars and uncertainty intervals in Figure 3). Importantly, there remains clear evidence for a reduction in incidence among women under all credible assumptions for the effect of ART. Our analysis of the impact of ART on the 2008 HIV prevalence levels showed that the effect was predominantly among women in the age group 25–49 years and among men 30 years and older. The increase of HIV prevalence due to ART (‘excess prevalence’) was 3.6 percentage points among women aged 30–34 years. The results argue strongly for the inclusion of antiretroviral (ARV) testing in national HIV survey protocols in order to enable a better informed interpretation of HIV prevalence data in the era of increasing ART coverage. The added costs are relatively modest in the context of the overall budget for a national cross-sectional survey.

Nationally-representative household-based surveys provide the most robust and reliable way to measure HIV prevalence in the general population [17]. Yet, biases in household surveys, in South Africa and other African countries, can potentially be caused by under-representing individuals not in households, e.g., mobile individuals or those living in institutions (prisons, army barracks, etc.). However, recent studies confirmed that the exclusion of non-household populations had only a minimal effect on the national estimates based on household survey populations [18], [19]. Besides successive cross-sectional surveys of the general population, the other major source of information on trends in HIV prevalence comes from prevalence estimates among women attending public antenatal services. Contrary to the observation among young women in the cross-sectional household surveys, the estimated national HIV prevalence among South African pregnant women remained stable over the past three years, 2006–2008 [1]. It would be informative to test whether there was a trend in HIV prevalence in the clinics that were included in all three years of surveillance, removing the possibility that the selection of different clinics has obscured a possible change.

However, extrapolations from antenatal data to the general population should be made with caution. Pregnant women seeking prenatal care at young ages are unlikely to be representative of young females in the general population. Teenage pregnancy in particular is an indicator for a socio-demographic and behavioural risk profile that greatly increases the risk of HIV infection. In many countries, including South Africa, antenatal estimates of prevalence at young ages are higher than estimates in the general population [20], [21]. Furthermore, prevalence trends observed in public antenatal clinics may not mirror trends in prevalence in the general population, if there are changes in age at first sex or shifting patterns of fertility with respect to age [22]. Indeed, any correlation between the degree of risk reduction among young women and the likelihood of pregnancy, potentially mediated by educational level or socio-economic background, could reduce an observed change in antenatal prevalence. Studies in Zambia [23] and Zimbabwe [24] have compared trends in prevalence measured in the general population with estimates among women attending local clinics and both found evidence that HIV prevalence trends in antenatal clinics underestimated trends in the general population, with the strength of this effect varying over time and by socio-economic risk group. We therefore believe the findings presented here are not necessarily contradictory to the findings of the antenatal surveys, but rather result from inherent differences associated with pregnant women served in public health clinics.

The application of the method we used to estimate HIV incidence for South Africa [4], [5] is limited to the general population at the national scale since there is a fundamental assumption about individuals in different age-groups being comparable [4]. For instance, 25 year-olds included in the 2005 survey and 28 year-olds included in the 2008 survey are drawn from the same population. This would not be the case for prevalence data obtained from sub-populations in which individuals may enter and exit as they age (such as injecting drug-users or sex workers). Therefore, an incidence analysis by specific risk groups or socio-behavioural factors associated with increased risk for HIV infection is beyond the scope of this method.

There are several potential factors that could have caused a reduction in incidence. As epidemics mature, incidence can decline as a natural course of the epidemic as those groups at most risk of infection become saturated with infection and die [25]. However, this may be unlikely to have had a major role in driving the incidence reduction among younger age-groups in an epidemic as established as in South Africa. The scale-up of ART may have the potential to reduce HIV incidence, since effective treatment reduces viral loads and, as a consequence, the infectiousness of infected individuals [26], [27]. However, since access to treatment has only increased significantly in recent years, it is expected that such an effect would take longer to develop and require higher levels of ART coverage for an extended period of time [16], [28].

The national surveys in 2002, 2005 and 2008 do not suggest substantial decreases in numbers of sexual partners, which have been associated with changes in the epidemic in Uganda and Zimbabwe [29], [30]. However, there is evidence from the surveys of a significant increase in condom use [6]. In 2002, 31% of men and women aged 15–49 used a condom at last sex, but by 2008 this had increased to 65%. The largest increases were among 15–24 year-old women (46% in 2002 to 73% in 2008) and 25–49 year-old men (27% to 56%), and earlier modelling work has shown that condom use in ‘cross-generational’ sexual partnerships (between older men and younger women) could have a particularly large effect on incidence among young women [31]. It not possible to quantify the extent to which these changes in reported sexual behaviour result from increasing social desirability bias [32], although one can argued that reporting on the number of sexual partners and condom use should change similarly if this bias was fully responsible for the observed changes. Further research is therefore required to clarify the ways in which risk of infection has been reduced, and the extent to which this can be attributed to programmes.

## Supporting Information

Technical Appendix S1 Method for adjusting HIV prevalence for the effect of antiretroviral therapy on survival.
(0.12 MB DOC)

Figure S1 Scale-up of antiretroviral treatment. The thick grey line shows the estimated numbers on ART in South Africa and the dashed yellow line shows the fitted second-order polynomial, used in the calculation for the trend in *Τ_γ_*. (Source: Department of Health, South Africa.)
(0.48 MB TIF)
