# Supplementary material for: A Decline in New HIV Infections in South Africa: Estimating HIV Incidence from Three National HIV Surveys in 2002, 2005 and 2008
Source: PLoS One. 2010 Jun 14;5(6):e11094. doi: 10.1371/journal.pone.0011094 (PMC2885415; doi:10.1371/journal.pone.0011094)
Supplement: Technical Appendix S1 — Method for adjusting HIV prevalence for the effect of antiretroviral therapy on survival. (0.12 MB DOC) [file pone.0011094.s001.doc]

***Technical Appendix***

***Method for Adjusting HIV Prevalence for the Effect of Antiretroviral Therapy on Survival***

To allow valid comparisons to be draw between the measured prevalence levels in 2002, 2005 and 2008, and to allow the previously described method of estimating mortality among those HIV infected but not on ART to be used correctly, we modify the observed HIV prevalence level in 2008 to remove the effect of antiretroviral therapy (ART) on survival. The adjusted 2008 prevalence estimate represents the expected prevalence level if treatment were not available. To do this, and following others [1], we subtract from the HIV prevalence measurement in 2008 the proportion of people in that survey who would not be alive if there were no treatment. This is not exactly the same as the number of people receiving treatment because, depending on the way in which treatment is initiated, some of these will have survived for some time even without treatment.

Formally, let $n_{i,y}$ and $p_{i,y}$ be, respectively, the size of the population and HIV prevalence of the $i^{th}$age-group in year $y$; $h_{i,y}$ be the proportion of the population in that age-group and year currently receiving ART; and, $\theta_{y}$be the proportion of people receiving treatment in that year that are alive due to the treatment (i.e. they would have died without treatment).

The corrected prevalence ($p_{i,y}*$) is calculated as:

$p_{i,y}*=p_{i,y}-\theta_{y}h_{i,y}$ … (1)

The fraction of those on treatment that are alive due to treatment ($\theta_{y}$) is estimated in the following way: let $q$be the mortality rate during the first years of treatment; $\alpha$be the mortality rate for those who need treatment but for whom treatment is not available; $S_{y,y+\delta}$be the number of people starting treatment between times $y$ and $y+\delta$. $T_{y}$is the number of people on treatment that year (the trend in $\hat{T}_{y}$ is inferred from available data on ART distribution). Thus,$S_{y,y+\delta}$can be calculated:

… (2)

Consider cohorts of infected individuals reaching the point at which treatment is/would be started in year $x$ - call this “cohort $x$”: if the cohort does not have treatment, the number surviving after $\tau$ years is: $S_{x,x+\delta}\exp(-\alpha\tau)$; but, if the cohort does have treatment, the number surviving is: $S_{x,x+\delta}\exp(-q\tau)$. Therefore, the number of people alive due to treatment in year $y$from cohort $x$is:

$e_{y,x}=S_{x,x+\delta}\left\{\exp\left(-q(y-x)\right)-\exp\left(-\alpha(y-x)\right)\right\}$ … (3)

If $y_{0}$ is the year when treatment first became available, then the total number of people alive in year $y$due to treatment is:

$E_{y}=\sum_{x=y_{0}}^{y} e_{y,x}$ … (4)

From this, we calculate:

… (5)

A second-order polynomial was fitted to the sentinel data on number on treatment, which was used to inform the trend in $T_{y}$(Figure S1).

For the point estimates, we used $\alpha=1$ to represent treatment being started usually one year before death would otherwise be expected [2,3,4] and, $q=0.1$ to represent 10% mortality rate during the first years of treatment [4,5]. We also assessed the uncertainty (reflected as error bars in Figure 3 and quoted uncertainty intervals in the main text) in the estimate of excess prevalence by making alternate assumptions about the ART adjustment: (i) that ART would have a greater effect on HIV prevalence (lower mortality on ART $q=0.05$); and, (ii) that ART would have a lesser effect on HIV prevalence (higher mortality on treatment $q=0.15$).

**References**

1. Hallett TB, Stover J, Mishra V, Ghys PD, Gregson S, et al. (2009) Estimates of HIV incidence from household-based prevalence surveys. AIDS 24: 147-152.

2. Egger M. Outcomes of Antiretroviral Treatment in Resource Limited and Industrialized Countries. 14th Conference on Retroviruses and Opportunistic Infections(<http://www.retroconference.org/2007/data/files/webpage_for_CROI.htm);> 2007; Los Angeles.

3. The eligibility for ART in lower income countries collaboration (2008) Duration from seroconversion to eligibility for antiretroviral therapy and from ART eligibility to death in adult HIV-infected patients from low and middle-income countries: collaborative analysis of prospective studies. Sex Transm Infect 84: i31-36.

4. Stover J, Johnson P, Zaba B, Zwahlen M, Dabis F, et al. (2008) The Spectrum projection package: improvements in estimating mortality, ART needs, PMTCT impact and uncertainty bounds. Sex Transm Infect 84 Suppl 1: i24-i30.

5. Braitstein P, Brinkhof MW, Dabis F, Schechter M, Boulle A, et al. (2006) Mortality of HIV-1-infected patients in the first year of antiretroviral therapy: comparison between low-income and high-income countries. Lancet 367: 817-824.
