# Supplementary figures and images for: A Decline in New HIV Infections in South Africa: Estimating HIV Incidence from Three National HIV Surveys in 2002, 2005 and 2008
Source: PLoS One. 2010 Jun 14;5(6):e11094. doi: 10.1371/journal.pone.0011094 (PMC2885415; doi:10.1371/journal.pone.0011094)

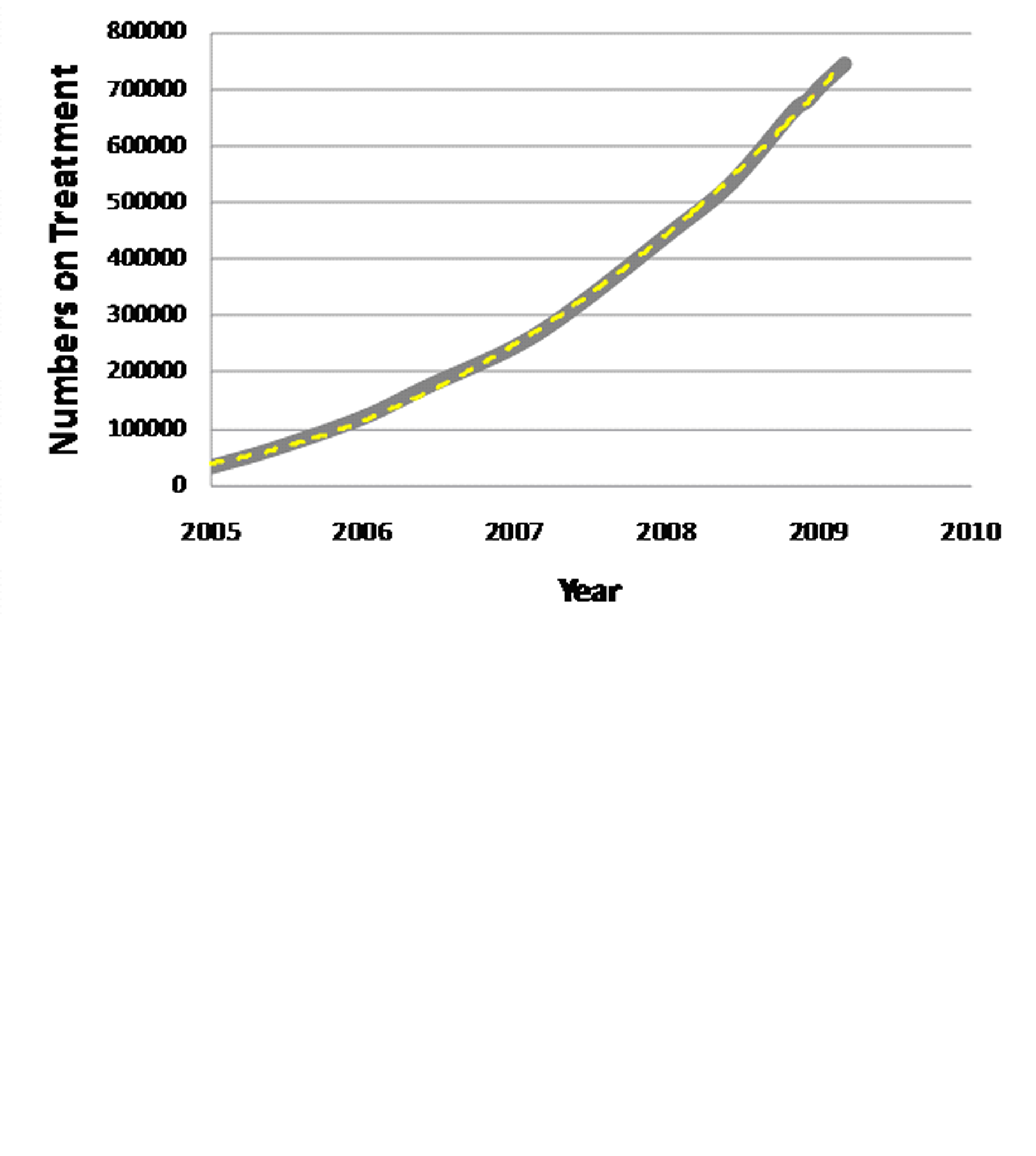

Supplement: Figure S1 — Scale-up of antiretroviral treatment. The thick grey line shows the estimated numbers on ART in South Africa and the dashed yellow line shows the fitted second-order polynomial, used in the calculation for the trend in Τγ. (Source: Department of Health, South Africa.) (0.48 MB TIF) [file pone.0011094.s002.tif]
